# Simultaneous Pheochromocytoma, Paraganglioma, and Papillary Thyroid Carcinoma without Known Mutation

**DOI:** 10.1155/2018/6358485

**Published:** 2018-10-14

**Authors:** Lorena Rasquin, Janna Prater, Jane Mayrin, Corrado Minimo

**Affiliations:** Einstein Medical Center, 5501 Old York Rd., Philadelphia, PA 19141, USA

## Abstract

**Background:**

Pheochromocytoma/paraganglioma is a rare tumor from neuroendocrine cells. 1/3^rd^ of cases have germline mutations. Papillary thyroid carcinoma (PTC) is a common neoplasm from follicular cells of the thyroid. We report a case of pheochromocytoma/paraganglioma and PTC with negative testing for common mutations.

**Case:**

32-year-old male with incidental liver mass during laparoscopy for acute appendicitis. His symptoms included abdominal pain and profuse axillary hyperhidrosis. MRI showed an 11x12x14 cm cystic and solid mass in right adrenal gland, and 3.4x2.9x3.8 cm mass in porta hepatis. Urine metanephrines was elevated. After preoperative alpha-blockade, patient underwent total right adrenalectomy. Pathology report confirmed diagnosis of pheochromocytoma. According to the Grading system for Adrenal Pheochromocytoma and Paraganglioma (GAPP), tumor's score was 9, indicating poorly differentiated tumor. Ki67 index 5% and S100 were negative. Postoperatively, plasma free metanephrines normalized but plasma free normetanephrines remained elevated. Based on this biochemical profile, presence of paraganglioma was suspected. CT showed 4.2x3.5 cm round soft tissue mass in porta hepatis which increased in size from previous MRI. Simultaneously, PET scan identified a 1.5 cm thyroid mass. Calcitonin level was normal. Fine-needle aspiration was consistent with PTC. Resection of the mass and total thyroidectomy were performed with confirmation of paraganglioma S100 positive and PTC. Normetanephrines decreased to 283 (<148 pg/mL); free metanephrines remained normal. Gene mutation of EGLN1, FH, KIF1B, MEN1, NF1, RET, SDHAF2, SDHC, SDHD, TMEM127, VHL, and SDHA was negative.

**Conclusion:**

Whether paraganglioma/pheochromocytoma/PTC combination is coincidental or resulted from an underlying unknown mutation cannot be excluded.

## 1. Introduction

Pheochromocytoma/paraganglioma is a rare tumor arising from neuroendocrine cells. Increasing numbers of susceptibility genes have been identified, and approximately 1/3^rd^ of cases have germline mutation. [1] Papillary thyroid carcinoma (PTC) on the other hand is a very common neoplasm arising from the follicular cells of the thyroid. Simultaneous presentation has been rarely described in literature. Few case reports and one four-case series were documented. Genetic studies have rarely been done, but were positive for SDHB gene.

Endocrine society clinical practice guideline recommended genetic testing using clinical driven diagnostic algorithms. They suggest testing for succinate dehydrogenase (SDH) mutations and that patients with metastatic disease should undergo testing for* SDHB *mutations. SDH gene encodes for portions of mitochondrial complex II which is a tumor suppressor gene involved in the electron transport chain and the tricarboxylic-acid (TCA) cycle. There are different subunit genes: SDHB, SDHC, SDHD, SDHAF2, and SDHA. Multiple paragangliomas have been identified in 66.9 percent of the SDHD mutation carriers; but malignant paragangliomas are more commonly seen in SDHB mutation carriers, 37.5 percent, as opposed to 3.1 percent of the SDHD, and none of the SDHC mutation carriers. [2] The main susceptibility genes have been identified as* SDHB*,* SDHD*,* VHL*, and* RET*. Patients with clinical predictive features should undergo genetic counseling.

**Figure 1:**
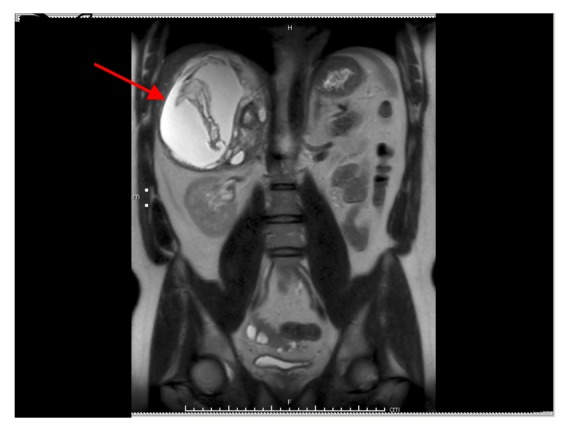
MRI abdomen shows 11 x 12 x 14 cm mixed cystic and solid mass centered in the right adrenal gland.

**Figure 2:**
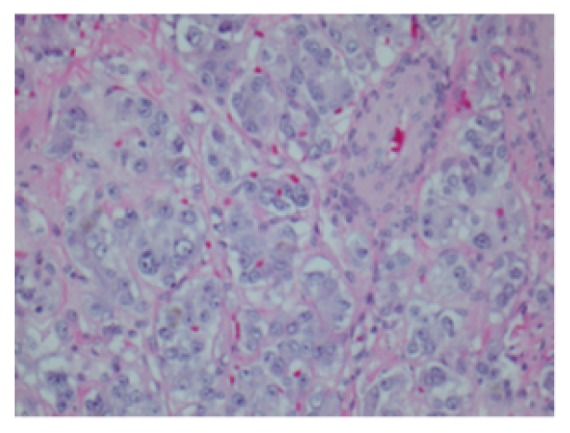
HE 40x pheochromocytoma.

**Figure 3:**
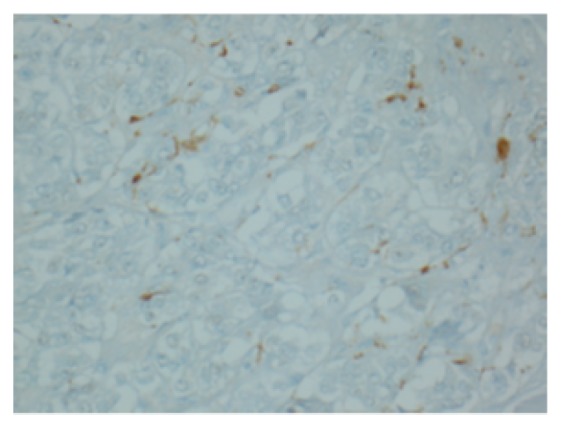
HC S100 sustentacular cells.

**Figure 4:**
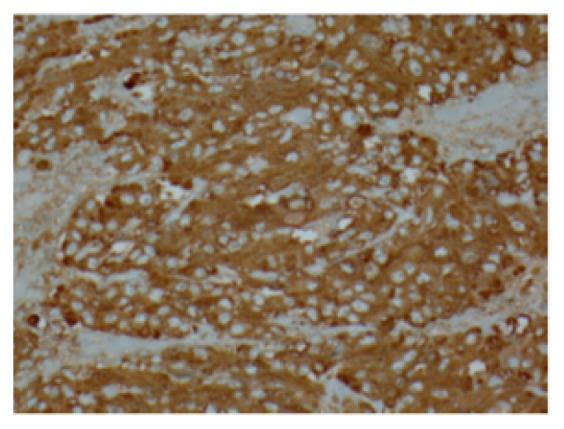
HC 40x chromogranin.

**Figure 5:**
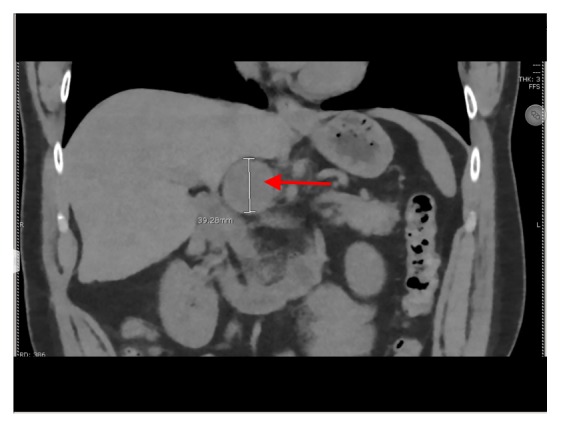
CT abdomen with porta hepatis paraganglioma.

We report a case of pheochromocytoma, paraganglioma, and PTC discovered subsequently with negative testing for most common mutations. Figures 1, 2, 3, 4, and 5 show MRI abdomen showing 11 x 12 x 14 cm mixed cystic and solid mass centered in the right adrenal gland, HE 40x pheochromocytoma, HC S100 sustentacular cells, HC 40x chromogranin, and CT abdomen with porta hepatis paraganglioma, respectively.

## 2. Case Report

32-year-old male without significant past medical history was found to have what appeared to be a liver mass during surgical exploration for an acute appendicitis. His symptoms preoperatively included intermittent abdominal pain and profuse episodic axillary hyperhidrosis. On examination, he was normotensive and did not show evidence of pallor, anxiety, or tachycardia. 24-hr urine metanephrine was 4339 (36-190 mcg/24 hr), normetanephrine 20025 (35-482 mcg/24 hr), and total metanephrines 24364 (116-695 mcg/24 hr). MRI of the abdomen showed an 11 x 12 x 14 cm mixed cystic and solid mass centered in the right adrenal gland and 3.4 x 2.9 x 3.8 cm mass in the region of the porta hepatis.

After preoperative alpha-blockade patient underwent total right adrenalectomy. Pathology report confirmed the diagnosis of pheochromocytoma. According to the Grading system for Adrenal Pheochromocytoma and Paraganglioma (GAPP) tumor's score was 9 [3], indicating poorly differentiated tumor with the presence of necrosis, irregular cell nest form, high cellularity, minimal capsular invasion, and adrenergic features. Ki67 index 5% and S100 were negative.

Postoperatively plasma free metanephrines had normalized at 31 (<57 pg/mL) but plasma free normetanephrine remained elevated at 1844 (<148 pg/mL). Based on this biochemical profile presence of paraganglioma was suspected. CT abdomen showed 4.2 x 3.5 cm round soft tissue mass in the region of the porta hepatis which slightly increased in size from previous MRI.

Simultaneously, positron emission tomographic scan identified a 1.5 cm thyroid mass. Calcitonin level was normal. Fine-needle aspiration of this nodule was consistent with papillary thyroid carcinoma. Resection of the porta hepatis mass and total thyroidectomy were performed with subsequent confirmation of paraganglioma and PTC, respectively. On surgical pathology paraganglioma was S100 positive. After the resection, level of plasma free normetanephrine had decreased to 283 (<148 pg/mL), and free metanephrine remained normal. Genetic studies, which included gene sequence changes and deletion/duplications of EGLN1, FH, KIF1B, MAX, MEN1, NF1, RET, SDHAF2, SDHC, SDHB, SDHD, TMEM127, VHL, and SDHA, were negative.

## 3. Discussion

Pheochromocytomas are rare tumors, widely described due to their serious morbidity and mortality rates. Most are sporadic tumor but 30% [4, 5] are inherited gene mutation which are described as part of a familial syndrome, which are associated with multiple neuroendocrine tumors. These gene mutations have been identified as part of Von Hipple Lindau syndrome, multiple endocrine neoplasia, neurofibromatosis type 1, and paragangliomas syndrome. However, papillary thyroid carcinoma has rarely been described in association with pheochromocytoma and from reported literature, when this association exists, different gene mutations have been identified; on the contrary is our case which was negative for all known mutations.

Germline mutations are five times more common among young patient, less than 45 years old; additionally multifocal or metastatic presentation has been associated with highest prevalence for gene mutation, four times higher when the tumor is extradrenal [5]. Genetic studies with patients presenting with these clinical predictors are highly recommended, despite the fact that these three risk factors were present in our patient, for which genetic studies were unrevealing.

From our review of literature of the studies found with simultaneous pheochromocytoma/paraganglioma and papillary thyroid carcinoma, the most comprehensive one was a four-case series analysis. It consisted of four female patients, one of them with synchronous PTC and PGL; 2 had heterozygous germline variants in SDHB and the four of them had -79 T>C CDKN1B gene polymorphism (3 homozygous and 1 in heterozygous state) [6]. This gene is not routinely tested in the panel for pheochromocytoma [7, 8]. The polymorphism -79>C CDKN1B was identified as a risk factor for developing the follicular variant of papillary thyroid carcinoma (FVPTC).

Whether paraganglioma/pheochromocytoma/PTC combination in our case is incidental or results from an underlying genetic predisposition is difficult to ascertain. Presence of unknown mutation cannot be excluded. The accelerated growth in genetic knowledge should prompt identification of additional susceptibility genes to be added in our genotyping panel in cases with high suspicion of mutation involvement such as presentation with multiple neoplasia.
